# A Single-Center Clinical Study to Evaluate Shenxiong Glucose Injection Combined with Edaravone in the Treatment of Acute Large-Area Cerebral Infarction

**DOI:** 10.1155/2021/9935752

**Published:** 2021-06-29

**Authors:** Zongqin Li, Xiaoxia Rong, Jun Luo, Tao Zeng, Pan Huang, Xuejie Xu

**Affiliations:** ^1^Department of Neurology, The Sichuan Mianyang 404 Hospital, No. 56 Yuejin Road, Mianyang, Sichuan, China; ^2^Department of Operations Management Division, The Sichuan Mianyang 404 Hospital, No. 56 Yuejin Road, Mianyang, China; ^3^Department of Thoracic Surgery, The Sichuan Mianyang 404 Hospital, No. 56 Yuejin Road, Mianyang, China; ^4^Pan Huang's department is Neurology, Deyang People's Hospital, 173 Taishan North Road, Deyang, China

## Abstract

**Objectives:**

To investigate the clinical efficacy and safety of Shenxiong glucose injection combined with edaravone in the treatment of acute large-area cerebral infarction.

**Methods:**

156 patients with acute large-area cerebral infarction admitted to our hospital from July 2015 to January 2017 were included in the analysis. The patients were randomly divided into experimental (78 cases) and control (78 cases) groups. Patients in the experimental group were given a 30 mg injection of edaravone in 100 ml of 0.9% sodium chloride solution by intravenous drip, twice a day within 30 minutes and a daily 200 ml injection of Shenxiong glucose by intravenous drip. Patients in the control group were given a 30 mg edaravone injection in 100 ml of 0.9% sodium chloride solution by intravenous drip, twice a day, and the drip was completed within 30 minutes. Patients in both groups were treated for 2 weeks. The levels of fibrinogen (FIB), D-dimer, interleukin 6 (IL-6), P-selectin (CD62P), and hypersensitive C-reactive protein (hs-CRP) were evaluated in the two groups of patients. Neurological disability was evaluated using the modified Rankin scale (mRS) and the neurological deficit score (National Institute of Health Stroke Scale, NIHSS). Adverse reactions to the treatments were also recorded.

**Results:**

No significant differences in age, gender, medical histories, and blood biochemical indices were observed between the two groups before treatment (*P* > 0.05). After treatment, the levels of FIB, D-dimer, IL-6, CD62P, and hs-CRP were significantly lower following treatment and compared to the control group (*P* < 0.05). Also, the mRS and NIHSS scores were significantly lower after treatment and compared with the control group (*P* < 0.05). The total effective rate of the treatment in the experimental group was significantly higher compared to the control group (*P* < 0.05). During the treatment period, no obvious adverse reactions were observed in the two groups of patients.

**Conclusions:**

In addition to the routine basic treatment of acute large-area cerebral infarction, the addition of Shenxiong glucose injection combined with edaravone injection can improve platelet aggregation and reduce inflammation by affecting P-selectin, D-dimer, and FIB. This treatment approach promotes the recovery of nerve defect function without obvious adverse reactions in patients with acute large-area cerebral infarction.

## 1. Introduction

Recent epidemiological studies have shown that cerebrovascular diseases have surpassed cancer as the number one cause of death in humans. The pathophysiological processes involved in the development of strokes are irreversible, and so the disease has a very high rate of disability that imposes a major public health burden in China [1]. Currently, arterial and venous thrombolysis and mechanical thrombectomy techniques have delivered improved outcomes for the majority of patients with acute cerebral infarction. However, for patients who cannot be treated with thrombolysis or mechanical thrombectomy, conservative medications are mainly adopted that include supportive therapy, improvement of ischemia tissue perfusion, and protection of brain cells [2, 3].

Edaravone is a free radical scavenger that can be used to protect the brain from injury by improving cerebral blood flow around the infarct and inhibiting lipid peroxidation [4]. The main components of Shenxiong glucose injection are Danshensu and ligustrazine hydrochloride which affects antiplatelet aggregation and dilation of the coronary artery and improves the microcirculation [5]. To date, there have been no reports on the efficacy and safety of these drugs in combination in the treatment of acute large-area cerebral infarction. This study is aimed at exploring the clinical efficacy, mechanism, and potential use of Shenxiong glucose injection combined with edaravone injection in the treatment of large-area cerebral infarction.

## 2. Information and Methods

### 2.1. Experiment Methods

The experiment method is a prospective randomized controlled study.

### 2.2. Inclusion Criteria

The inclusion criteria are as follows: (1) acute cerebral infarction caused on the basis of arteriosclerosis, diabetes, and hypertension, that is, TOAST classification is aortic atherosclerosis; (2) onsettime < 48 d; (3) patients with first onset; and (4) all patients were diagnosed with large-area cerebral infarction by MRI or CT examination. Large area is defined as the largest slice diameter of cerebral infarction measured by MRI, or CT examination is greater than 5.0 cm, or the infarction spreads to two brain lobes, or the spread of cerebral infarction is larger than 1/2 or 2/3 of the area on the same side.

### 2.3. Exclusion Criteria

The exclusion criteria are as follows: (1) severe dementia, (2) severe mental symptoms or mental illness who cannot cooperate, (3) severe liver and kidney failure, (4) heart disease, (5) combined severe infection, and (6) those who are allergic to the drugs used in this study.

### 2.4. Research Subjects Selected

**Figure 1:**
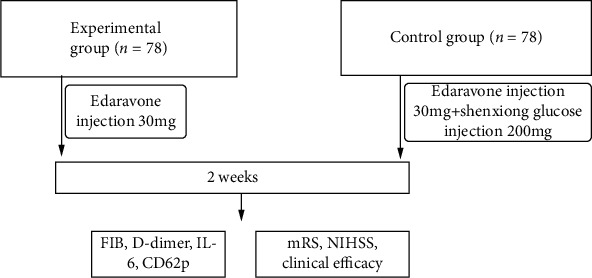
The experimental flow chart.

This study included 156 patients admitted to the Sichuan Mianyang 404 Hospital from July 2015 to January 2017 with acute large-area cerebral infarction. All patients met the diagnostic criteria for cerebral infarction in the Acute Ischemic Stroke Treatment Guidelines [6]. Using the random table method, all patients were randomized into experimental (78 cases) and control groups (78 cases). The experimental group consisted of 42 males and 36 females, aged between 50 and 80 years with an average of 61.74 (±4.67) years. The experimental group consisted of 44 males and 34 females, aged between 52 and 81 years with an average age of 62.82 (±4.83) years. No significant differences in the general patient characteristics were observed between the two groups (*P* > 0.05). The time from onset to admission for blood collection in both groups ranged from 1 to 48 hours with an average time of 18.25(±1.14) hours. The research protocol was reviewed and approved by the medical ethics committee of the hospital, and all participants were recruited under written informed consent document. The experimental flow used in the study is shown in Figure 1.

### 2.5. Treatment Methods

After admission, the two groups of patients were dehydrated to reduce cerebral edema and treated with antiplatelet aggregation and other basic treatments. In addition to the basic treatment, patients in the experimental group were given a 30 mg edaravone injection in 100 ml of 0.9% sodium chloride solution by intravenous drip, twice a day within 30 minutes and a daily injection of 200 ml of Shenxiong glucose. Patients in the control group were given a 30 mg injection of edaravone in 100 ml of 0.9% sodium chloride solution by intravenous drip, twice a day within 30 minutes, and treatment was continued for two weeks. The Shenxiong glucose injection was obtained from the Guizhou Yibai Injection Pharmaceutical Co., Ltd. (Approval Number: National Medicine Zhunzi H52020703, specification 100 ml/bottle). The edaravone injection was obtained from the Sinopharm Guorui Pharmaceutical Co., Ltd. (Approval Number: National Medicine Standard H20080056, specification 20 ml : 30 mg).

### 2.6. Specimen Collection and Testing

All blood samples were collected from the anterior cubital venous blood on an empty stomach within 24 hours after admission. The plasma was separated and extracted by the EDTA anticoagulant tube. The serum was separated and extracted from the tube without anticoagulant and stored in a freezer at -20°C. The serum levels of CD62p were determined by flow cytometry (American Applied Biosystems). A flow cytometry kit was used for analysis (Shanghai Xuanhao Technology Co., Ltd) according to the manufacturer's instructions. Serum D-dimer levels were detected using a magnetic bead method (normal value = 0‐1 *μ*g/ml). Serum hs-CRP levels were determined using an immunoturbidimetric method with a Deling's BN-Prospec special protein instrument for detection. IL-6 levels were determined by ELISA using test kits purchased from the R&D company production.

### 2.7. Observation Indices

The neurological deficit scores of the two groups were obtained before and after treatment which included the mRS and NIHSS scores. The levels of P-selectin (CD62P), D-dimer, hs-CRP, IL-6, and FIB were evaluated before and after treatment in the two groups. The occurrence of adverse reactions was also recorded in the two groups.

### 2.8. Efficacy Evaluation [6]

The efficacy of treatment was defined as follows: (1) cure: when the degree of disability was zero, and the NIHSS score was reduced by ≥90% compared to that before treatment; (2) significantly effective: when the degree of disability was one to three, and the NIHSS score was reduced by 46%-<90% compared to that before treatment; (3) effective: when the degree of disability was one to three, and the NIHSS score was reduced by >18%-45% compared to before treatment; (4) ineffective: when the symptoms of the patient and signs of disease did not change significantly or worsened before and after medication, and when the NIHSS score was reduced by ≤18% compared to before treatment; and (5) totally effective: when the cure + markedly effective + effective.

### 2.9. Statistical Analysis

The SPSS 21.0 software was used for statistical analysis. Measurement data were expressed as *x* ± *s* and analyzed using a *t*-test. Counting data were expressed as the number of cases and analyzed using a *x*^2^ test. Grade data were expressed as the number of cases or rates and analyzed using a rank-sum test. *P* values < 0.05 were used to indicate statistical significance.

## 3. Results

### 3.1. Comparison of General Data in the Two Patient Groups before Treatment

No significant differences were detected in the age, past medical histories, and blood biochemical examinations between the two groups before treatment (*P* > 0.05). The patient characteristics are summarized in Table 1.

There was no significant difference in age, past medical history, and blood biochemical examinations between the two groups before treatment (*P* > 0.05), indicating that the two groups are comparable (Table 1).

### 3.2. Comparison of CD62P, D-Dimer, IL-6, FIB, and hs-CRP Levels before and after Treatment in the Two Patient Groups

Before treatment, the two groups of patients had no significant differences in the levels of CD62P, D-dimer, hs-CRP, FIB, and IL-6 (*P* > 0.05). After treatment, the levels of these biomarkers were all significantly lower than before treatment (*P* < 0.05) with larger decreases observed in the experimental group. The data are summarized in Table 2.

### 3.3. Comparison of the Neurological Deficit Scores between the Two Groups before and after Treatment

Before treatment, no significant differences were observed between the mRS and NIHSS scores of the two groups of patients (*P* > 0.05). After treatment, the mRS and NIHSS scores of the two groups of patients were significantly lower than before treatment. The level of improvement in each score in the experimental group was significantly higher than in the control group (*P* < 0.05). The data are summarized in Table 3.

### 3.4. Comparison of Clinical Efficacy between Two Groups of Patients

After the combined treatment with the two drugs, the total effective rate of the experimental group was 89% which was significantly higher than the 77% total effective rate observed in the control group (*P* < 0.05). The data are summarized in Table 4.

### 3.5. Comparison of Adverse Reactions between the Two Groups of Patients

During the entire treatment period, no adverse reactions such as skin rash, nausea, vomiting, fever, or headache were observed in the patients. No obvious abnormalities were detected by electrocardiogram, routine blood and urine analysis, and liver and kidney function tests.

## 4. Discussion

Acute cerebral infarction is caused by obstructions of brain blood supply that lead to cerebral ischemia, hypoxic necrosis, and corresponding neurological deficits. In China, acute cerebral infarction has surpassed cancer as the leading threat to human health. Studies have confirmed that cerebral infarction is closely related to platelet aggregation [7, 8] which is caused by platelet activation [9]. P-selectin (CD62P) is a glycoprotein that has a molecular mass of 140,000 and is mainly distributed on *α* particles in stationary platelets. When platelets are activated, *α* particles quickly fuse with the platelet membrane and are released to allow redistribution of CD62p on the platelet surface [10]. The concentration of CD62p reaches a peak 10 minutes after platelet activation and can be used as a specific marker for evaluating platelet activation and thrombosis [11]. Animal experiments have shown that the expression of CD62P increases significantly 1 hour after cerebral infarction and reaches a peak at 8 to 24 hours that can last for 3 to 5 days. Also, CD62P can promote the activation of granulocytes to aggravate inflammation and coagulation reactions which can promote the formation of thrombus [12].

The fibrinolytic function in the body also changes when cerebral infarction occurs [13, 14]. D-dimer is a specific degradation product formed by fibrin cross-linking and redissolving. D-dimer reflects the fibrinolytic function in the body. After thrombosis, the level of D-dimer in the body changes, and so it can be used as a biomarker in clinical practice [15] for the early diagnosis of cerebral infarction and to evaluate thrombolytic effects. FIB is a coagulation protein that participates in platelet aggregation and the coagulation processes and can also promote the occurrence of cerebral infarction [16].

After cerebral infarction, the focal area is composed of a necrotic center and an ischemic penumbra. Damage to brain cells located in the ischemic penumbra can be reversed, and these cells can potentially be protected from damage [17]. Brain protective drugs include free radical scavengers, opioid receptor blockers, and excitatory amino acid receptor blockers. However, neuroprotective agents have made significantly less progress compared to reperfusion therapy [18, 19]. The American Stroke Treatment Development Roundtable (STAIR) reviewed the experience and lessons of the past 30 years and put proposed future areas for neuroprotective research and development including more adequate preclinical trials, multitarget protection, and combined reperfusion therapy [20, 21].

Under the guidance of STAIR principles, two exciting developments in neuroprotective agents have been reported in the past two years: firstly, the ESCAPE-NA1 study in which a PSD-95 inhibitor was obtained in the subgroup of combined perfusion therapy positive result [22, 23] and, secondly, TASTE research which uses multitarget protection as an important theoretical principle [24]. Multitarget protection is aimed at the cascade reaction after cerebral ischemia. This complex pathophysiological process results in damage to brain cells and is driven by energy metabolism disorders, hypoxic depolarization around the infarction, calcium overload, excitatory amino acid toxicity, oxidative stress damage, and the inflammatory response and other signaling pathways [25]. Multiple pathways may be involved at different points during the development of brain damage, and a single drug targeting a single mechanism is unlikely to effectively control the disease. Edaravone is a free radical scavenger that has a 3-methyl-1-phenyl-2-pyrazoline-5-one structure with lipophilic groups and a good ability to cross the blood-brain barrier [26]. In the central nervous system, edaravone mainly protects nerve cells by scavenging oxygen free radicals, inhibiting lipid peroxidation, regulating inflammatory factors, and inhibiting apoptosis [27–31].

Shenxiong glucose injection is a compound preparation that is composed of Danshensu, ligustrazine, glucose, and glycerin. Studies have confirmed that Danshensu and ligustrazine hydrochloride can resist platelet aggregation, reduce blood viscosity, improve brain microcirculation, and protect vascular endothelial function. Studies have found that Shenxiong glucose injection can improve neuronal tolerance to ischemia, promote the recovery of damaged neurons, reduce infarct volume, and improve prognosis [32].

The results of this study show that the FIB, D-dimer, IL-6, P-selectin, and hs-CRP levels and the mRS and NIHSS scores in the two groups of patients decreased after a two-week treatment compared to before treatment and the control group. The decreases were more obvious in the experimental group (*P* < 0.05) suggesting that Shenxiong glucose injection combined with edaravone can improve the symptoms of neurological impairment in patients. Also, the effective rate in the experimental group was significantly higher than the control, and no obvious adverse reactions were observed during treatment.

In summary, Shenxiong glucose injection combined with edaravone is a safe and effective treatment for acute large-area cerebral infarction. However, the data reported in this study requires further validation due to the small sample size and investigation of a single-center study. Also, the follow-up was relatively short, and further studies should include larger patient cohorts in a multicenter study.

## Figures and Tables

**Table 1:** Comparison of general information of the two groups of patients.

Item	Experimental group (*n* = 78)	Control group (*n* = 78)	*P*
Age (years)	60.74 ± 4.67	62.82 ± 4.83	0.15
Sex (M/F)	40/38	41/37	0.52
Smoke (*n*, %)	28 (35.89%)	30 (38.46%)	0.48
Hypertension (*n*, %)	18 (23.07%)	21 (26.92%)	0.39
Diabetes (*n*, %)	11 (14.10%)	12 (15.38%)	0.62
SCr (*μ*mol/l)	52.24 ± 6.47	51.81 ± 6.20	0.67
UA (*μ*mol/l)	354.85 ± 37.25	361.23 ± 38.96	0.29
TC (mmol/l)	4.82 ± 0.41	4.90 ± 0.42	0.23
TG (mmol/l)	1.23 ± 0.25	1.26 ± 0.28	0.48
HDL-C (mmol/l)	0.95 ± 0.23	0.96 ± 0.22	0.78
LDL-C (mmol/l)	2.88 ± 0.43	2.87 ± 0.41	0.88
AST/ALT	0.72 ± 0.22	0.73 ± 0.21	0.77
BMI (kg/m^2^)	22.51 ± 1.21	22.73 ± 1.24	0.26
Coronary heart disease	12 (15.38)	13 (16.67)	0.64
Atrial fibrillation	8 (10.25)	9 (11.53)	0.62

BMI: body mass index; TG: triglyceride; TC: total cholestrol; HDL-C: high density lipoprotein-cholestrol; LDL-C: low high density lipoprotein-cholestrol.

**(a):** 

	CD62P (ng/l)		D-dimer (*μ*g/ml)		Hs-CRP (mg/l)	
	Bef Tre	Aft Tre	Bef Tre	Aft Tre	Bef Tre	Aft Tre
Experimental group	36.70 ± 2.45	34.23 ± 2.21^∗^	2.99 ± 0.71	0.85 ± 0.22^∗^	10.65 ± 1.22	8.56 ± 0.85^∗^
Control group	37.45 ± 2.49	36.56 ± 2.32^∗^	3.01 ± 0.75	1.23 ± 0.41^∗^	11.02 ± 1.33	9.75 ± 1.12^∗^
*T*	1.89	6.42	0.17	7.21	1.81	7.47
*P*	0.59	0.00	0.86	0.00	0.07	0.00

**(b):** 

	FIB (ng/l)		IL-6 (pg/ml)	
	Bef Tre	Aft Tre	Bef Tre	Aft Tre
Experimental group	3.85 ± 0.86	3.43 ± 0.56^∗^	14.27 ± 1.25	10.23 ± 1.02^∗^
Control group	3.65 ± 0.78	3.27 ± 0.41^∗^	13.89 ± 1.45	11.45 ± 1.15^∗^
*T*	1.52	2.03	1.75	7.00
*P*	0.13	0.04	0.08	0.00

Compared with before treatment, ^∗^*P* < 0.05. Bef Tre: before treatment; Aft Tre: after treatment.

**Table 3:** Comparison of neurologic impairment degree scores between 2 groups before and after treatment.

	NIHSS		mRS	
	Before Tre	Aft Tre	Bef Tre	Aft Tre
Experimental group	38.26 ± 2.78	24.53 ± 1.7	3.89 ± 0.85	1.84 ± 0.45
Control group	37.79 ± 1.50	28.98 ± 1.89	3.85 ± 0.82	2.21 ± 0.56
*T*	1.30	15.13	0.29	0.00
*P*	0.19	0.00	0.76	0.00

Bef Tre: before treatment; Aft Tre: after treatment.

**Table 4:** Comparison of clinical efficacy rate between 2 groups.

Item	Cure	Significantly effective	Effective	Ineffective	Total effective (%)
Experimental group	14	18	38	9	89%
Control group	10	15	35	18	77%^#^

Compared with the experimental group, ^#^*P* < 0.05.

## Data Availability

All data, models, and code generated or used during the study appear in the submitted article.
